# Functional Resistance to Recurrent Spatially Heterogeneous Disturbances Is Facilitated by Increased Activity of Surviving Bacteria in a Virtual Ecosystem

**DOI:** 10.3389/fmicb.2018.00734

**Published:** 2018-04-11

**Authors:** Sara König, Anja Worrich, Thomas Banitz, Hauke Harms, Matthias Kästner, Anja Miltner, Lukas Y. Wick, Karin Frank, Martin Thullner, Florian Centler

**Affiliations:** ^1^Department of Ecological Modelling, The UFZ – Helmholtz Centre for Environmental Research, Leipzig, Germany; ^2^Department of Environmental Microbiology, The UFZ – Helmholtz Centre for Environmental Research, Leipzig, Germany; ^3^Institute of Environmental Systems Research, University of Osnabrück, Osnabrück, Germany; ^4^Department of Environmental Biotechnology, The UFZ – Helmholtz Centre for Environmental Research, Leipzig, Germany; ^5^German Centre for Integrative Biodiversity Research (iDiv) Halle-Jena-Leipzig, Leipzig, Germany

**Keywords:** stability, microbial ecosystem service, simulation model, ecological modeling, fragmentation, resilience, biomass distribution, bacterial degradation

## Abstract

Bacterial degradation of organic compounds is an important ecosystem function with relevance to, e.g., the cycling of elements or the degradation of organic contaminants. It remains an open question, however, to which extent ecosystems are able to maintain such biodegradation function under recurrent disturbances (functional resistance) and how this is related to the bacterial biomass abundance. In this paper, we use a numerical simulation approach to systematically analyze the dynamic response of a microbial population to recurrent disturbances of different spatial distribution. The spatially explicit model considers microbial degradation, growth, dispersal, and spatial networks that facilitate bacterial dispersal mimicking effects of mycelial networks in nature. We find: (i) There is a certain capacity for high resistance of biodegradation performance to recurrent disturbances. (ii) If this resistance capacity is exceeded, spatial zones of different biodegradation performance develop, ranging from no or reduced to even increased performance. (iii) Bacterial biomass and biodegradation dynamics respond inversely to the spatial fragmentation of disturbances: overall biodegradation performance improves with increasing fragmentation, but bacterial biomass declines. (iv) Bacterial dispersal networks can enhance functional resistance against recurrent disturbances, mainly by reactivating zones in the core of disturbed areas, even though this leads to an overall reduction of bacterial biomass.

## Introduction

Microbial ecosystems provide a variety of services ranging from the cycling of elements (e.g., C or N), to the recycling of nutrients and the degradation of contaminants. In particular, this includes the biodegradation of organic compounds in terrestrial environments. These are, however, often exposed to disturbances which can (directly or indirectly) affect microbial population dynamics and associated ecosystem functions. For instance, disturbance events may inhibit bacterial growth and activity, alter competitive ability, increase bacterial mortality, or reduce substrate availability (de Ruiter et al., 2002; Botton et al., 2006; Shade et al., 2012). Especially when disturbance events recur repeatedly, the cumulative effects may impact the long-term stability with respect to structure and function (Ho et al., 2015). It is, however, unknown to which extent microbial ecosystems maintain their biodegradation performance under such recurring disturbances and which factors govern this response. The ability to stay essentially unchanged despite disturbances is referred to as resistance (Grimm and Wissel, 1997). Specifically, we define the ability of the system to sustain its function under recurrent disturbances as functional long-term resistance.

Although the resistance of microbial ecosystems has recently gained increasing attention, many studies focused on structural aspects such as the response of community composition to disturbances (Allison and Martiny, 2008; Bressan et al., 2008; van der Zaan et al., 2010; Manzoni et al., 2012; Kim et al., 2013; Jechalke et al., 2014). Functional resistance, in contrast, has less often been addressed although its importance has been acknowledged (Tobor-Kaplon et al., 2005; Berga et al., 2012; Griffiths and Philippot, 2013).

Therefore, it is crucial to gain mechanistic understanding of how microbial ecosystem structure and functions respond to recurrent disturbances. We know from general ecology and the theory of complex adaptive systems that ecological systems can cope with change and maintain their functions, if the change is not too intense (Harrison, 1979; Pimm, 1984; Ho et al., 2015). We already know from previous studies that spatial aspects can be particularly important for the stability of microbial ecosystems (Altermatt et al., 2011; Baho et al., 2012; Shade et al., 2012; König et al., 2017). Especially in highly heterogeneous systems such as soil, disturbances are often characterized by a spatial pattern of occurrence. Only few disturbance types in the terrestrial environment occur homogeneously like a change in temperature (Rousk et al., 2012; Jurburg et al., 2017). Most disturbances affect an expanded, heterogeneous area which is often linked to the specific pore network structure, like draining or flooding events, salinization, or toxic substances entering the subsurface (Edwards, 2002; Evans and Wallenstein, 2012; Smith et al., 2016). Furthermore, it is also widely accepted that mechanisms enhancing connectivity (i.e., the degree to which an environment allows organisms to disperse among different resource patches) support resource uptake, regeneration, survival and, hence, resistance in heterogeneous habitats. In microbial ecosystems, this connectivity may be mediated by dispersal networks facilitating bacterial movement such as fungal hyphae (Kohlmeier et al., 2005; Wick et al., 2007; Furuno et al., 2010; Banitz et al., 2011b; Knudsen et al., 2013; Ellegaard-Jensen et al., 2014; Simon et al., 2015; Otto et al., 2016). Hence, it is of interest whether, when, and to which extent such dispersal networks can benefit the functional resistance of biodegradation. However, the relationship between functional resistance and the (spatial and temporal) characteristics of disturbances, and the influence of dispersal networks on such long-term resistance of microbial ecosystems has not yet been systematically investigated.

The experimental investigation of the spatiotemporal dynamics of microbial processes in terrestrial environments is challenged by the small process scales and the high complexity of these ecosystems in general. An alternative and powerful investigation approach is the use of microbial simulation models (Banitz et al., 2012; Stolpovsky et al., 2012; Ebrahimi and Or, 2014; Esser et al., 2015; König et al., 2017). The aim of these models is not to describe a microbial community and its surrounding environment in full detail but to simplify it adequately such that selected processes and their impacts on the entire system’s dynamics can be studied in isolation. This approach allows for simulating and analyzing situations which are not easily implementable in experimental work, such as highly spatially heterogeneous environments or frequent identical perturbations. Such simulation models were successfully applied in previous studies to address various topics such as biogeochemical processes (Schäfer et al., 1998; Wirtz, 2003; Centler et al., 2010; King et al., 2010), bioclogging (Thullner and Baveye, 2008; Brovelli et al., 2009), functional dynamics during litter decay in soils (Kaiser et al., 2014), microbial dormancy (Stolpovsky et al., 2011), bacterial mobility in different environments (Banitz et al., 2013; Gharasoo et al., 2014; Centler and Thullner, 2015), or nitrogen compound transformations (Costa et al., 2006; van de Leemput et al., 2011).

Here, we use a modeling approach to systematically analyze the dynamic response of biodegradation (as an exemplary ecosystem function) and bacterial biomass to recurrent disturbances of different characteristics (frequency and spatial pattern). Our spatially explicit simulation model *eColony* comprises the processes of bacterial resource consumption, growth, dispersal, and disturbance events leading to the death of bacterial cells (König et al., 2017). It also allows for the simulation of bacterial dispersal networks. The model serves as a tool for analyzing and comparing the dynamic response of biodegradation performance and bacterial biomass to different regimes of recurrent disturbances at two spatial scales: the overall performance/bacterial biomass of the centimeter-scale system and the local biodegradation performance/bacterial biomass in individual habitats (i.e., individual model grid cells on the millimeter-scale). To our knowledge, no experimental study exists on the long-term response of microbial functions to recurring, heterogeneously distributed disturbances so far. Therefore, we aim to close a gap with our modeling study and improve the understanding of the dynamic response to recurrent disturbance events. In particular, we explore and characterize (i) the ranges of recurrent disturbances under which the microbial ecosystem maintain a sufficient biodegradation performance, (ii) the loss of this performance when the disturbance regimes are more severe, (iii) the relationship between the response of biodegradation performance and bacterial biomass to recurrent disturbances, and (iv) the potential of dispersal networks to enhance long-term resistance against recurrent disturbances.

## Materials and Methods

### Simulation Model eColony

#### Model Description

We applied the spatiotemporally explicit, population-based numerical model (*eColony*) for assessing the functional resistance of bacterial populations to disturbance regimes varying in spatial and temporal occurrences. The model developed by König et al. (2017) describes bacterial growth, dispersal, and substrate degradation (e.g., biodegradation of an organic compound) as well as the diffusive transport of the dissolved substrate with a set of reaction–diffusion equations. It is an extension of an established model which had been parameterized to laboratory experiments with motile flagellated bacteria on agar plates (Banitz et al., 2011a, 2012). The model operates on a two dimensional circular domain with a diameter of 88 mm, reflective boundaries, and consists of rectangular habitats of 1 mm^2^ size each. The model equations are solved using a finite difference method implemented in the programming language Delphi.

The following processes are included in each 1-min simulation step: substrate uptake by bacteria, uptake allocation to energy-demanding tasks, bacterial growth and reproduction, bacterial dispersal, and substrate diffusion.

**FIGURE 1:**
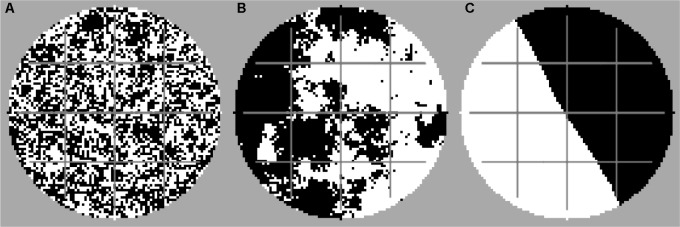
Examples of disturbance patterns with high fragmentation **(A)** (*H* = –1), moderate fragmentation **(B)** (*H* = 0.5), and no fragmentation **(C)** (*H* = 2). Each scenario was simulated with and without dispersal networks. Black, disturbed area; white, undisturbed area; gray, dispersal network.

For simulating networks facilitating quick dispersal of the bacteria (mimicking the “fungal highway” effect of mycelial networks, e.g., Kohlmeier et al., 2005), corridors of habitats with a very high bacterial diffusivity are implemented. If this model feature is activated, the bacteria can disperse much faster in the habitats covered by the dispersal networks. The networks’ structure is fixed (cf. **Figure 1**, gray lines), and no other fungal processes such as mycelial growth or decay are considered.

The model allows for a permanent substrate input representing a supply by dissolution (cf. Keymer et al., 2006; Centler et al., 2011). Assuming an area-wide permanent substrate input that allows substrate to accumulate within a habitat up to a maximum substrate concentration *C_s,max_*, substrate is refilled in each time step depending on the difference between the maximum concentration *C_s,max_* and the current substrate concentration within a particular habitat *C_s_*, multiplied by the input rate parameter λ (cf. Eq. 2).

#### Equations

Considering the depicted extensions the dynamics of bacterial colony growth and substrate degradation are described by the extended reaction–diffusion equations:

$$\frac{\partial C_{x}}{\partial t}=\nabla (D_{x}\left(C_{x},C_{s}\right)\nabla C_{x})+\left(q(C_{s})Y_{G}-a-d\left(C_{x},C_{s}\right)\right)C_{x},$$

$$\frac{\partial C_{s}}{\partial t}=D_{s}\nabla^{2}C_{s}+\lambda (C_{s,\max}-C_{s})-q\left(C_{s}\right))C_{x},$$

where *C_x_* and *C_s_* are the concentrations of bacteria (g_x_ mm^-2^) and substrate (g_s_ mm^-2^), *D_x_* and *D_s_* are the diffusion coefficients of bacteria and substrate (mm^2^ h^-1^), *q* is the specific substrate uptake rate of bacteria (g_s_ g_x_^-1^ h^-1^), which is calculated according to $q=q_{\max}\frac{C_{S}}{C_{S}+K_{S}}$ with *q_max_* as maximum specific uptake rate (g_s_ g_x_^-1^h^-1^) and *K_s_* as half-saturation constant (g_s_ mm^-2^). *Y_G_* is the growth yield coefficient (g_x_ g_s_^-1^), *a* the specific maintenance rate (h^-1^), *d* is the specific dispersal cost, expressed as biomass decrease rate (h^-1^), and λ the substrate input rate parameter (h^-1^). Note that both *D_x_* and *d* vary depending on substrate availability (cf. Banitz et al., 2011a, 2012). An overview of used parameter values is given in **Table 1**.

**Table 1:** Base set of model parameter values and initial conditions.

Parameter/ State variable	Symbol	Value	Unit^a^	Source
Maximum specific growth rate	*μ_max_*	0.347	h^-1^	Banitz et al., 2016
Specific maintenance rate	a	0.0003	h^-1^	Banitz et al., 2011a
Growth yield	*Y_G_*	0.6	g_x_g_s_^-1^	Banitz et al., 2011a
Maximum substrate uptake rate	*q_max_*	0.578	g_s_g_x_^-1^h^-1^	q_max_ = $q_{\max}=\frac{\mu_{\max}+a}{Y_{G}}$
Half-saturation constant	*K_s_*	4.439E-07	g_s_mm^-2^	Banitz et al., 2011a
Maximum bacterial diffusion coefficient	*D_x,max_*	0.212	mm^2^h^-1^	Banitz et al., 2012
Maximum bacterial diffusion coefficient along dispersal networks	*D^dn^_x,max_*	144	mm^2^h^-1^	Banitz et al., 2011a
Substrate diffusion coefficient	*D_s_*	2.326	mm^2^h^-1^	Zhang and Fang, 2005
Substrate input rate	*λ*	0.24	h^-1^	Keymer et al., 2006
Initial bacterial concentration	*C_x_*^∗^	2.366E-4	g_x_mm^-2^	undisturbed reference state
Initial substrate concentration	*C_s_*^∗^	3.847E-11	g_s_mm^-2^	undisturbed reference state


*^*a*^g_*x*_, grams of dry biomass; g_*s*_, grams of substrate.*

#### Model Assumptions

Our population-based model simulates a single population of cells with identical properties, and does not consider any adaptation processes. The effective growth rate *μ_eff_* depends on substrate concentration *C_s_* in the particular habitat: the more substrate is available, the more is consumed and used for growth, up to the maximum growth rate *μ_max_*. Active bacterial mobility is considered by a bacterial diffusion coefficient *D_x_* which also depends on the local substrate concentration (Banitz et al., 2012). No bacterial movement takes place if the extant substrate concentration is below the fixed maintenance rate *a*, in which case all available substrate is used for maintenance. If the substrate concentration is higher than the maintenance rate, but lower than the maximum dispersal cost *d_max_*, the bacterial diffusion coefficient is linearly increasing with increasing substrate uptake. The bacteria can thus disperse faster if more substrate is available, up to the maximum bacterial diffusion coefficient *D_x,max_*. On dispersal networks, a higher maximum bacterial diffusion coefficient *D^dn^_x,max_* is applied. With these interactions of the processes growth and dispersal, we mimic bacterial chemotaxis toward areas of higher substrate concentrations (Wei et al., 2011; Banitz et al., 2012).

We assume a constant water saturation in the model corresponding to conditions that restrict (but not inhibit) bacterial dispersal. Substrate diffusion is implemented as an undirected diffusive process with constant diffusion coefficient *D_s_*. Other processes (e.g., advective transport or sorption) were not considered. All parameter values were directly taken from or fitted in accordance with experimental work using agar as a medium (**Table 1**).

### Disturbance Scenarios

Based on the substrate input, we calculated the (spatially homogeneous) steady state as an undisturbed reference state (**Table 1**) in which the substrate input per time step matches exactly the bacterial uptake which, in turn, exactly satisfies their maintenance demand. Thus the bacterial population in undisturbed conditions continuously receives sufficient energy for survival but not for growth and, consequently, remains constant.

For each disturbance scenario, initial concentrations of bacterial biomass and substrate were adjusted to the values of the undisturbed reference scenario (**Table 1**). Pulse disturbance events were introduced as shock events in which bacterial biomass concentrations are severely reduced within a certain disturbance area. In each disturbed habitat, the bacterial population is reduced by multiplying biomass with a factor of 10^-9^ (i.e., surviving biomass of 2.366e^-13^ g mm^-2^ which is equivalent to one surviving bacterial cell after the first disturbance). The bacteria in habitats in the undisturbed area are not directly affected by the disturbance events.

We used the midpoint displacement algorithm for creating random fractal landscapes with the fragmentation parameter *H* and proportion *p* (e.g., Saupe, 1988) for generating the disturbance patterns. For all simulations, the proportion *p* of the disturbed area was set to 50% of the simulated domain. The fragmentation parameter *H* was varied between -1 and 2 in steps of 0.2 for representing a span between the extreme situations of “highly fragmented” (*H* = -1) and “non-fragmented” (*H* = 2) disturbance patterns. Example patterns with different degrees of fragmentation are shown in **Figure 1**. Within one simulation run, the disturbance events occurred always with the same spatial disturbance pattern, i.e., always the same set of habitats was affected by the disturbances (assuming that the applied disturbance area depends on physical soil characteristics, e.g., given by the spatial arrangement of a pore network system in soil). However, we always performed at least 10 different simulation runs for each scenario, to randomly generate ensembles of disturbance patterns coinciding in the degree of fragmentation but differing in the explicit spatial configuration. This allows us to tackle the question of whether the explicit spatial configuration or certain aggregated spatial characteristics of the disturbance pattern such as the degree of fragmentation are decisive for the functional resistance.

Disturbance events were repeated at a constant frequency, i.e., after a constant disturbance return interval. The disturbance return interval length in the different simulations varied from 10 to 150 hours (again with at least 10 replicates for each scenario). Thus, regimes of recurrent disturbance events are operationalized via the spatial pattern and return interval of their occurrence in our modeling framework.

### Analysis

**FIGURE 2:**
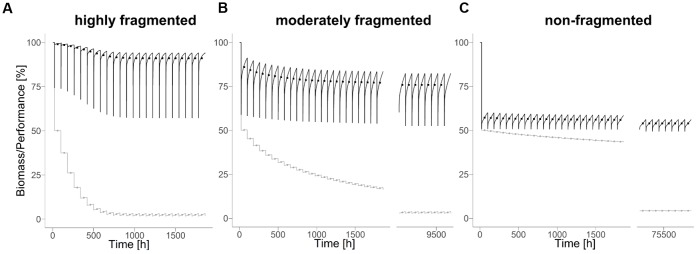
Biodegradation performance (black) and bacterial biomass (gray) under disturbance regime with highly **(A)** (*H* = –1), moderately **(B)** (*H* = 0.5), and non-fragmented **(C)** (*H* = 2) disturbance pattern and a disturbance return interval of 80 hours. Points indicate mean values during corresponding disturbance return interval.

We simulated each scenario until reaching a constant mean level of biodegradation performance and bacterial biomass between consecutive disturbance events (**Figure 2**). This final quasi-steady state was the basis for subsequent analyses of the biodegradation performance and bacterial biomass under recurrent disturbances, calculated for each habitat separately and for the whole system. The spatiotemporal dynamics were examined by classifying each habitat’s mean biodegradation performance over a time span equivalent to the disturbance return interval in relation to the undisturbed reference state (denoted as 100%) with a change of 5% points considered to be significant: (i) “enhanced” habitats, if the respective local biodegradation performance is above 105%, (ii) “unchanged” habitats, if the performance is between 95% and 105%, (iii) “reduced” habitats, if the performance is between 5% and 95%, and (iv) “inhibited” habitats, if the performance is below 5%.

To further assess the influence of the spatial configuration of the disturbance events on biodegradation performance and bacterial biomass we calculated the mean distance between habitats in the disturbed and the respective nearest habitat in the undisturbed area Δ:

$$\Delta =\frac{1}{\left|DA\right|}\Sigma_{(i,j)\in DA}\min_{(k,l)\in UA}\sqrt{{\left(k-i\right)}^{2}+{\left(l-j\right)}^{2},}\,$$

Here, *DA* is the set of disturbed and *UA* the set of undisturbed habitats, *|DA|* is the number of disturbed habitats, *k* and *l* are Cartesian coordinates of undisturbed habitats and *i* and *j* Cartesian coordinates of disturbed habitats. Based on Δ values for each simulation run, we analyzed the relation between this metric of the spatial disturbance pattern and the respective resistance of the biodegradation function and the bacterial biomass to the disturbance.

For analyzing the relevance of the disturbance patterns’ explicit spatial configuration we calculated the standard deviation (SD) of the biodegradation performance and bacterial biomass values for all disturbance patterns belonging to the same disturbance ensemble, with SD = 0 indicating no relevance.

## Results

### Influence of Spatial Disturbance Pattern

In all scenarios analyzed, the overall biodegradation performance and the bacterial biomass approach a constant mean level over the course of time (**Figure 2**). In the following, this new quasi-steady state levels of biodegradation performance and bacterial biomass serves as a measure for long-term resistance. The levels of both measures depend on the degree of fragmentation of the disturbance pattern. Interestingly, biodegradation performance increases with increasing fragmentation of the disturbances, whereas bacterial biomass decreases. However, the time needed for bacterial biomass to approach a quasi-steady state is generally longer than for the biodegradation performance.

In all scenarios, the performance of the just-disturbed system significantly exceeds 50% of the performance of the undisturbed system. This is surprising as 50% of the simulation area is repeatedly disturbed with almost complete removal of bacterial biomass in the disturbed area. It gives rise to the hypothesis that the disturbed area still contributes to the biodegradation function and/or that the undisturbed area exhibits a higher performance than in absence of disturbances. To test this hypothesis, we analyzed the spatiotemporal dynamics in terms of the mean biodegradation performance of each habitat in quasi-steady state under disturbances compared to its performance in the undisturbed reference state (cf. section “Analysis” for the classification scheme).

**FIGURE 3:**
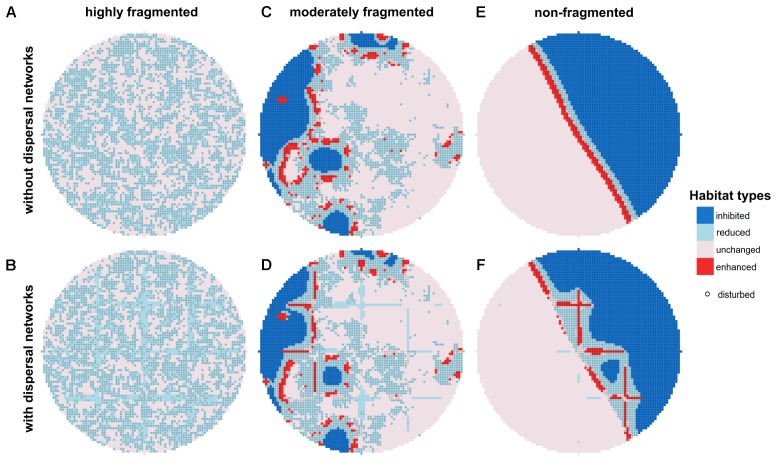
Mean biodegradation performance in quasi-steady state without **(A,C,E)** and with dispersal network **(B,D,F)** under recurrent disturbances with a disturbance return interval of 80 h. Three different degrees of fragmentation of the disturbance pattern are shown: highly **(A,B)** (*H* = –1), moderately **(C,D)** (*H* = 0.5), and non-fragmented **(E,F)** (*H* = 2). Habitats within the disturbance area (50% of total area) are marked with gray circles.

Analyzing exemplary results for a disturbance return interval of 80 hours (i.e., disturbance events occur every 80 hours), the spatial distribution of the local (habitat-specific) quasi-steady state biodegradation performance reveals heterogeneity in the biodegradation performance in response to the underlying spatial disturbance pattern (**Figure 3**). Evidently, several disturbed habitats can be classified as “reduced,” i.e., they still show biodegradation performance above 5%. The amount of these habitats decreases with the degree of fragmentation of the disturbance pattern (fewer light blue habitats from left to right in **Figure 3**).

Under highly fragmented disturbances, biodegradation takes place in both disturbed and undisturbed habitats, as is indicated by the two distinct habitat types displayed: in the undisturbed area, most habitats are classified as “unchanged” and only a few as “reduced,” whereas in the disturbed area all habitats are classified as “reduced” (**Figure 3A**). In this scenario, there are no habitats with “enhanced” biodegradation activity, and also no “inhibited” habitats. In presence of dispersal networks, however, the amount of “reduced” habitats slightly increases at the expense of “unchanged” habitats in the undisturbed area (**Figure 3B**).

In scenarios where the disturbance is moderately fragmented, the picture is different (**Figure 3C**). Here, clusters of “enhanced” habitats occur at the interface between the disturbed and the undisturbed area. Those clusters are surrounded by “unchanged” habitats in the undisturbed area and “reduced” habitats in the disturbed area. However, the biodegradation performance in many disturbed habitats is classified as “inhibited.” Dispersal networks increase the amount of active habitat types in the disturbed area due to the enhanced bacterial dispersal which allows bacteria from the undisturbed area to quickly colonize also more distant disturbed habitats resulting in increased biodegradation performance in those habitats (**Figure 3D**).

Under non-fragmented disturbances, almost the entire disturbed area consists of “inhibited” habitats except for locations close to the interface to the undisturbed area, where “reduced” and “enhanced” habitats are present (**Figure 3E**). However, due to the short overall length of the interface between disturbed and undisturbed area, the number of these habitats is low and, consequently, the biodegradation performance is mainly provided by the undisturbed area. Similar to the scenario with moderately fragmented disturbance patterns, the presence of dispersal networks causes an increase of the amount of active (i.e., “reduced”) habitats in the disturbed area (**Figure 3F**).

**FIGURE 4:**
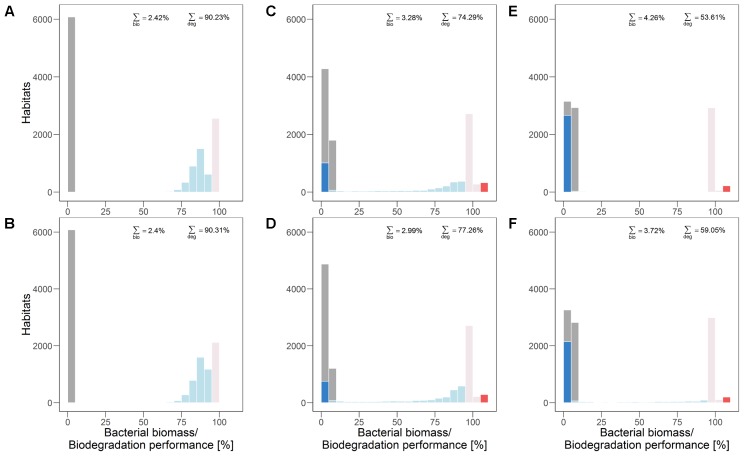
Distribution of bacterial biomass (gray) and biodegradation performance (colors according to classification, cf. section “Analysis”) in quasi-steady state without **(A,C,E)** and with dispersal networks **(B,D,F)** under recurrent disturbances with a disturbance return interval of 80 h. Three different degrees of fragmentation of the disturbance pattern are shown: highly **(A,B)** (*H* = –1), moderately **(C,D)** (*H* = 0.5), and non-fragmented **(E,F)** (*H* = 2).

The total biodegradation performance decreases with a decreasing degree of fragmentation of the disturbance pattern, whereas bacterial biomass decreases with increasing fragmentation (**Figure 4**, cf. Supplementary Figure S1). In scenarios with dispersal networks, the total biodegradation performance under moderate and non-fragmented disturbances increases while bacterial biomass decreases compared to the scenario without dispersal networks.

Under highly fragmented disturbances also the total biodegradation performance is highest (approximately 90%). The contribution of all habitats keeps the total biodegradation performance at this high level (**Figure 4A**). Dispersal networks cause a reduction of the amount of “unchanged” habitats in the undisturbed area which is, however, compensated for by higher biodegradation performance in other “reduced” habitats (**Figures 4A,B**). Hence, the total biodegradation performance is almost unaffected. Bacterial biomass is evenly distributed on a very low level (0–5%) and similar for the scenarios without and with dispersal networks.

Under moderately fragmented disturbances the overall biodegradation performance (approximately 74%) is mainly provided by “unchanged” habitat types, but also by “enhanced” and “reduced” habitats (**Figure 4C**). In the presence of dispersal networks the overall biodegradation performance is enhanced due to the shrinking fraction of “inhibited” habitats in the disturbed area while bacterial biomass is slightly reduced (**Figures 4C,D**).

For non-fragmented disturbance patterns the total biodegradation performance is even much lower (approximately 54%) than for moderately fragmented disturbance patterns. This is due to the higher amount of “inhibited” habitats (**Figure 4E**). Again, biodegradation performance is enhanced (by more than 5%) due to dispersal networks as they decrease the number of “inhibited’ (i.e., inactive) habitats. Bacterial biomass is again reduced in presence of dispersal networks (**Figures 4E,F**).

Thus, the degree of spatial fragmentation of the recurrent disturbances influences both, the occurrence and the spatial distribution of habitat types. The microbial ecosystem’s overall biodegradation performance in quasi-steady state (i.e., the functional long-term resistance) increases with disturbance fragmentation (from right to left in **Figure 4**). In contrast, the bacterial biomass in quasi-steady state decreases with fragmentation.

**FIGURE 5:**
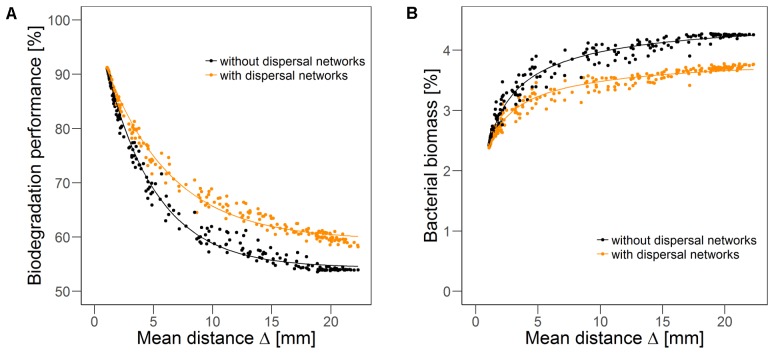
Biodegradation performance **(A)** and bacterial biomass **(B)** in quasi-steady state as indicators of long-term resistance over mean distance Δ between disturbed and nearest undisturbed habitats in scenarios without (gray) and with (orange) dispersal networks under recurrent disturbances with a disturbance return interval of 80 h. Disturbance fragmentation was varied between *H* = –1 and *H* = 2 in steps of 0.2, with 20 simulation runs with different disturbance patterns per step. Solid line indicates non-linear fit (**A**: R^2^ = 0.994 without dispersal networks, R^2^ = 0.993 with dispersal networks, **B**: R^2^ = 0.977 without dispersal networks, R^2^ = 0.966 with dispersal networks).

Systematic stepwise variation of the degree of fragmentation (increasing the value of fragmentation parameter *H* from -1 to 2 in increments of 0.2, cf. section “Disturbance Scenarios”) results in continuous variation of the mean distance from each disturbed to its nearest undisturbed habitat Δ. The quasi-steady state biodegradation performance (**Figure 5A**) is negatively correlated, while the bacterial biomass (**Figure 5B**) is positively correlated to this mean distance. For short mean distances, i.e., disturbed habitats are close to the undisturbed area, the mean overall biodegradation performance is high. With increasing mean distance, the overall biodegradation performance declines exponentially. With dispersal networks, the overall biodegradation performance follows a similar decline, but the performance is higher than without dispersal networks, except for very low mean distances where biodegradation performance is very high also without dispersal networks and they do not provide any further benefit. Thus, the difference in the performance with and without dispersal networks increases with mean distance until reaching a level of approximately 5% of the initial biodegradation performance at a mean distance of 12 mm. For longer mean distances, the benefit in the biodegradation performance remains almost constant. The overall bacterial biomass exhibits the opposite trend (**Figure 5B**). With increasing mean distance the bacterial biomass increases until approaching saturation (at a level slightly above 4%). In the presence of dispersal networks, bacterial biomass is lower for most scenarios, although again the difference disappears for small distances. These observations show that, although the spatial distributions of bacterial biomass and biodegradation activity continuously change over time, the resulting mean biodegradation performance and bacterial biomass strongly correlate with a single spatial measure of the recurrent disturbance pattern: the mean distance between disturbed and undisturbed habitats.

### Influence of Disturbance Return Interval

**FIGURE 6:**
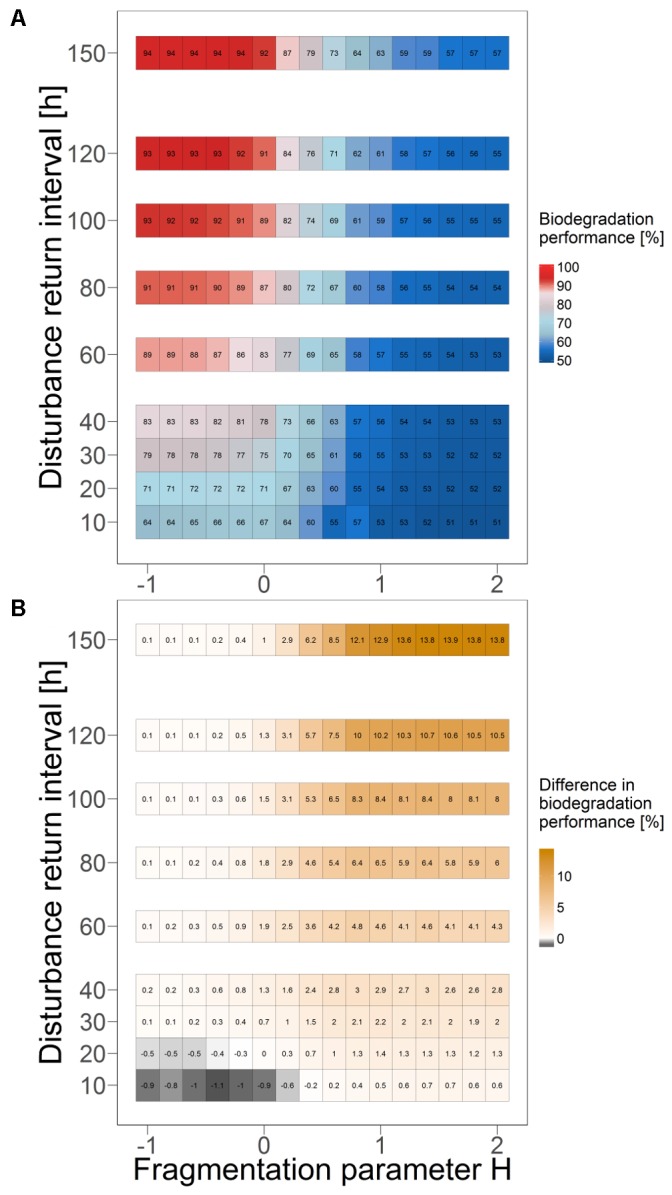
Mean biodegradation performance in quasi-steady state as an indicator of functional resistance for different disturbance return intervals and degrees of fragmentation. Boxes show mean values of 10 independent simulation runs. **(A)** Without dispersal networks. **(B)** Change in mean performance for scenarios with and without dispersal networks. Positive differences indicate a better performance with dispersal networks.

Varying the value for the disturbance return interval between 10 and 150 hours indicates that the revealed positive correlation between mean overall biodegradation performance in quasi-steady state and the degree of fragmentation of the disturbance pattern is qualitatively independent of the return interval length. This is also true for the negative correlation between mean overall bacterial biomass and degree of fragmentation (**Figure 6A** and Supplementary Figure S3). However, results also show that the microbial ecosystem maintains a total biodegradation performance close to that in the undisturbed case (high functional resistance), if the return interval length exceeds a certain threshold (sufficiently rare disturbance events). The value of this threshold decreases with an increasing degree of fragmentation. With a return interval length below this threshold (too frequent disturbance events), the overall biodegradation performance declines and deviates increasingly from the performance level in the undisturbed case (lower functional resistance). These results were obtained without (**Figure 6A**) and with dispersal networks (Supplementary Figure S2). Bacterial biomass also increases with less frequent disturbance events, although this effect is more distinct for higher fragmented disturbances (Supplementary Figures S3, S5, S8, S9).

Moreover, the disturbance return interval also affects the degree of spatial differentiation in the habitat-specific biodegradation performances (Supplementary Figures S6, S7, S10, S11). Under highly frequent disturbance events (i.e., a disturbance return interval of 20 hours), biodegradation performance is most heterogeneously distributed in the system as all kinds of habitat types (from “enhanced” to “inhibited’) emerge in considerable amounts. Longer disturbance return intervals result in more evenly distributed biodegradation performance as the extreme habitat types (“enhanced” and “inhibited”) get less likely. The overall biodegradation performance benefits from dispersal networks only under specific disturbance regimes (**Figure 6B**). Those disturbances are moderately or less fragmented and close to the critical return intervals, below which the microbial ecosystem’s functional resistance declines. Outside this zone, there is no considerable benefit from dispersal networks, and for some disturbance regimes the mean biodegradation performance is even slightly decreased in their presence. The latter was particularly observed for very short disturbance return intervals (10 and 20 hours, **Figure 6B**).

## Discussion

### Determinants of Functional Long-Term Resistance Against Recurrent Disturbances

Our study indicates that the modeled microbial ecosystems can maintain a high biodegradation performance under recurrent disturbances, as long as the affected areas are sufficiently spatially fragmented and the disturbance events are not too frequent. A previous study showed that the functional recovery time after a single disturbance event gets shorter with an increasing degree of fragmentation of the disturbance pattern and that spatial processes (substrate diffusion and bacterial dispersal) are key for this faster recovery (König et al., 2017). The current study shows that these factors are also controlling the functional long-term resistance under recurrent disturbance events. Thus, functional long-term resistance is linked to the functional recovery after a particular disturbance event before the next event occurs. Although the bacterial biomass eventually declines under such disturbances to a very low (but stable) level, it remains sufficient for maintaining the functional performance at a high level which can surprisingly exceed by far the proportion of the overall area that is disturbed. Moreover, the biodegradation performance and bacterial biomass show opposite responses to the spatial fragmentation of the recurrent disturbances.

In particular, a high spatial fragmentation of the disturbance pattern results in a higher overall length of the interface between disturbed and undisturbed areas. This allows for easier distribution of unconsumed substrate from disturbed to undisturbed habitats on the one hand, and for dispersal of bacterial cells from undisturbed to disturbed habitats on the other hand, leading to a high biodegradation performance under recurrent disturbances. This result is in line with experimental studies, showing that dispersal processes can stabilize a system (Cadotte and Fukami, 2005; Low-Decarie et al., 2015; Worrich et al., 2016). For instance, Cadotte and Fukami (2005) demonstrated that dispersal can decrease the time needed for a microbial community to reach its equilibrium. We further found that a high functional resistance comes along with a rather even distribution of the biodegradation performance among the different habitats. In this case, all effects of spatial heterogeneity caused by the disturbance events are substantially dampened in the course of time between the disturbance events. In contrast, with a lower spatial fragmentation of the disturbance pattern these spatial exchange processes get more limited, and thus the emerging biodegradation performance is reduced, but the long-term bacterial biomass is higher. Here, the spatial clustering of undisturbed habitats creates “safety areas” which are also the major contributor of the remaining functional performance. These areas prevent too many bacteria from dispersing to the repeatedly disturbed, resource-rich habitats where they, in consequence, will be removed during the subsequent disturbance event. In terms of the bacterial biomass, we have thus observed “edge effects” which are well known in ecology (Bascompte and Sole, 1996; Oliver et al., 2015; Brinck et al., 2017). More edges (e.g., a longer interface between disturbed and undisturbed areas) result in a higher risk for the bacteria to be affected by the disturbance. Various studies indicate that spatial aggregation enhances the stability of microbial communities due to a higher species richness in the center of such biofilms compared to the edges (Kanavillil et al., 2014), increased horizontal gene transfer (Madsen et al., 2012), cooperation between neighboring bacterial cells (Pande et al., 2015), or a higher cell density (Butler et al., 2010).

Both biodegradation performance and bacterial biomass were found to be correlated with the mean distance between disturbed and undisturbed habitats. The correlation was negative for biodegradation performance and positive for bacterial biomass due to the described opposed effects of the spatial fragmentation of the disturbance patterns. These correlations indicate that a summarizing spatial metric can condense the spatial characteristics that are relevant for the functional resistance, while the pattern’s particular explicit spatial configurations are of minor relevance.

### Reduced Functional Long-Term Resistance Comes Along With Spatial Differentiation of Biodegradation Performance

Once the coping capacity of the microbial ecosystem is exceeded, the overall biodegradation performance declines. Moreover, the microbial ecosystem responds with a spatial differentiation of the biodegradation performance in the individual habitats, ranging from completely “inhibited” to “enhanced” compared to the habitat-specific local biodegradation performance in the undisturbed reference case.

With decreasing degree of fragmentation, overall biodegradation performance declines as was already discussed. But even as the overall biodegradation performance declines, the local biodegradation performance in specific habitats at the interface between disturbed and undisturbed areas can rise in response to recurrent disturbance events. This is caused by increased substrate availability and consumption in these habitats due to a diffusive substrate influx from neighboring disturbed habitats (where consumers are less abundant). Moreover, the high spatial clustering of the undisturbed habitats causes “safety areas” with a high distance to the disturbed area where the bacterial biomass is less affected by the recurring disturbance events. These “safety areas” can act as a source of bacterial biomass dispersing toward the disturbed area. Thus, the local performance increases in specific habitats at the interface of undisturbed and sufficiently large clusters of disturbed habitats (cf. **Figure 3**). Both this spatial differentiation and the overall decline in biodegradation performance get stronger when disturbance events become more frequent or less fragmented. This also reveals that the temporal structure of the disturbance events recurring on the same area governs the emerging spatial distribution of biodegradation performance, i.e., it has a pattern-forming effect on activity.

The long-term resistance of microbial ecosystems under recurrent disturbances due to recolonization from undisturbed source habitats was studied previously both with experiments and modeling by analyzing structural responses to disturbances (Altermatt et al., 2011; Baho et al., 2012; Galic et al., 2012; Shade et al., 2012). For instance, Altermatt et al. (2011) showed in an experimental study that a connection between disturbed and undisturbed habitats increases the recovery of bacterial biomass after disturbances. Within our modeling study, this is similar for the biodegradation performance, but the bacterial biomass decreased with a higher connectivity between disturbed and undisturbed habitats (e.g., higher fragmentation of disturbance pattern). This difference may be explained by the fact that in the study of Altermatt et al. (2011), the high biomass recovery after disturbances was driven by the disturbed habitats, where dispersal from connected undisturbed habitats (immigration) counteracted the effects of disturbances, as it is the case for functional resistance here. However, in our system, this dispersal also has a substantial negative effect on the remaining bacterial biomass in the undisturbed habitats (emigration), which leads to the low resistance of bacterial biomass, but is not the case in the system studied by Altermatt et al. (2011). In the experimental work of Baho et al. (2012), the presence of refuges has proven to increase the structural recovery after disturbances, which is comparable with our “safety areas” enhancing the long-term resistance of bacterial biomass under recurrent disturbances of low spatial fragmentation.

We have shown that the biodegradation performance is reduced under recurrent disturbances, but still remains at a level that is clearly above that of the undisturbed habitats alone. This means that the disturbed habitats substantially contribute to the overall biodegradation performance, although the bacterial biomass here is quite low. The spatially explicit model analysis revealed that the habitats mainly responsible for this additional activity depend on the disturbance regime. As long as the disturbance return interval is not too short, a substantial part of the disturbed habitats contribute significantly to the overall biodegradation performance, while, additionally, undisturbed habitats along the border to the disturbed area even increase their activity. Hence, both disturbed as well as undisturbed habitats contribute to observed biodegradation activity above 50%. However, if the disturbance return interval is too short, the majority of disturbed habitats remain completely inactive. In these cases, the additional biodegradation performance is mostly achieved by increased activity in the undisturbed habitats along the border to the disturbed area. This is a clear indication for a combined effect of temporal and spatial characteristics of the recurrent disturbances on biodegradation performance and its spatial differentiation.

### Relevance of Dispersal Networks

Dispersal-mediated enhancements of connectivity are beneficial for regeneration and stabilization in many ecological contexts (Moloney and Levin, 1996; Loreau et al., 2003; Pe’er et al., 2011; Steiner et al., 2011). Steiner et al. (2011), for instance, showed that enhanced dispersal increases the stability and biodiversity of competing zooplankton species under the influence of fluctuating pH conditions.

Our results reveal that dispersal networks can substantially enhance the functional resistance and partly buffer the adverse impacts of a given disturbance regime, provided the disturbed area is moderately or non-fragmented and the return interval exceeds a certain minimum. Bacteria can disperse much faster along the networks, can reach habitats relatively far inside the disturbed area, and can thus better access and degrade the substrate in this disturbed area. Under different conditions, dispersal networks do not markedly increase functional resistance because biodegradation performance recovers quickly already without them (high spatial fragmentation of disturbances). This result is supported by an experimental study showing that dispersal networks increase the resistance against osmotic stress if the bacteria are heterogeneously distributed, but not if they are homogeneously distributed (Worrich et al., 2016). In other scenarios of our study, dispersal networks are not beneficial because they cannot help with overcoming the negative impact of quickly recurring, spatially clumped disturbance events (moderate or no spatial fragmentation of disturbances and short return intervals). Here, marginal benefits in functional long-term resistance may even come along with losses in long-term bacterial biomass (Supplementary Figures S11d,g vs. S10d,g). This is due to an accelerated dispersal of bacteria out of the undisturbed into the disturbed area and removal of these bacteria by disturbance events before they can substantially improve biodegradation performance. The general effects of bacterial dispersal networks are again rather independent of the explicit spatial configuration of the disturbance pattern, as is confirmed by the low values of the SD in functional performance under dispersal networks for almost all different disturbance regimes (Supplementary Figure S4). Exceptions are observed for disturbances with moderate fragmentation: here, the SD is higher as the explicit spatial configuration of the disturbance pattern dictates the mean distance of disturbed to the next undisturbed habitat which is an important factor for the functional resistance (cf. **Figure 5**).

Our spatially explicit model analysis also reveals that the benefit from dispersal networks for the long-term resistance of biodegradation is mostly due to the maintenance of activity in otherwise inactive habitats in the interior of the disturbed area. A comparable activation effect was observed in an experimental work showing that mycelia can stimulate bacterial activity by transporting nutrients and water, and with this, increase stability in stressed environments (Worrich et al., 2017). In our simulations, this effect is rather moderate and restricted to the surroundings of the dispersal networks. It can also slightly reduce the local biodegradation performance in certain undisturbed habitats, as bacteria with direct access to the dispersal networks leave these habitats. Hence, the networks lead to a shift in distributions of local biodegradation performance, which may both increase or reduce spatial heterogeneity, but do not necessarily lead to an enhancement of overall biodegradation performance.

### Model Limitations and Potentials

We used a spatially explicit, population-based model describing the dynamics of bacterial growth, dispersal and substrate biodegradation under recurrent disturbances in a simplified manner. This approach reduces the complexity, but captures ubiquitous and essential processes of natural systems. In particular, we assumed that disturbance events recur with a constant spatial pattern, reducing the bacterial biomass in the disturbed area without direct effects in the undisturbed area or on bacterial dispersal (without or along dispersal networks). We also excluded potential substrate limitation and intra-population or community variation in physiological traits of bacteria which may affect functional stability (e.g., Fernandez et al., 2000; Griffiths et al., 2001; Botton et al., 2006).

In case of diffusive transport in microbial systems, as in our study, specific pore space geometries have only little influence on spatiotemporal bacterial distribution (Gharasoo et al., 2014) and the use of simplified models focusing on the interaction of specific processes helps understanding the dynamics of microbial systems (King et al., 2010; Banitz et al., 2011b; Centler et al., 2011; Stolpovsky et al., 2011; Kaiser et al., 2014). Using the model to systematically vary the disturbance regimes’ spatial fragmentation and return interval length, we identified general phenomena that may have a high relevance for microbial ecosystem functioning under recurrent disturbances. Particularly, the spatial explicitness of the simulation model enabled us to analyze the spatiotemporal biodegradation dynamics on different scales and to assess the influence of the disturbance pattern’s spatial configuration on functional long-term resistance.

When determining the influence of dispersal networks, also a variation of their spatial configuration may be of interest (Banitz et al., 2016). However, dispersal networks applied in the present study are a good representation of well-connected mycelial networks that cover the entire system. Networks that are sparser or have less coverage may be less beneficial, whereas denser networks may lead to even higher improvements. It is unlikely that dispersal networks with different spatial configurations would have significantly altered our findings regarding their potential for improved functional resistance to recurrent disturbances, and the spatial and temporal disturbance characteristics determining this potential.

Following a “virtual lab” approach, we were able to simulate a wide range of spatially heterogeneous disturbance events occurring in arbitrary but constant return intervals which is hardly realizable in laboratory experiments. Analyzing both the biodegradation performance and the bacterial biomass was important for understanding the relationship between both indicators for resistance. Thus, this study underlines the advantages of comprehensive simulation modeling for gaining insights into microbial ecosystems and associated functions.

### Implications for Natural Systems

The high sensitivity of biodegradation performance to the spatial configuration of disturbance patterns may be especially relevant for different soil types with various pore size distributions and pore connectivity. In soil, these spatial environmental characteristics may considerably influence the area affected by disturbances such as drought events, the release of chemicals toxic to the bacteria, or increasing salt concentrations. This may lead to rather fragmented or highly clumped disturbance patterns. Thus, the degree of functional resistance to recurrent disturbances is likely also dependent on the soil type. For instance, a soil system consisting of well-mixed particles may be highly fragmented and, thus, more functionally resistant to recurrent disturbances. Contrarily, a soil system where different particles are aggregated in different areas may form a pore network in which disturbances occur in larger patches, whereas the remaining areas are not directly affected by the disturbances. As we have shown, this may lead to a decrease in overall functional resistance, although it may increase bacterial biomass. This conclusion is underpinned by a recent review identifying pore connectivity as one of the key indicators for assessing soil functions (Rabot et al., 2018). However, the type of disturbance should also be considered. The observed impact of the disturbance pattern’s spatial configuration on functional resistance applies to disturbances that affect some but not all pores, such as toxic chemicals released to the system or drastic drought events decreasing the water potential. In case of disturbance events that affect the whole system homogeneously, such as temperature fluctuations, the particular pore network structure is likely to be less relevant.

Similarly, our simulation study shows that the biodegradation benefits provided by bacterial dispersal networks also depend on the spatial configuration of the disturbance pattern. Thus, also the potential of such networks to enhance biodegradation performance under recurrent disturbances may vary for different soil types. We suggest that further studies with the focus on microbial functional resistance to disturbance regimes in soil systems should involve the influence of different pore structures. Importantly, we observed decoupled and in many cases even opposed dynamic responses of bacterial biomass and biodegradation performance to recurring disturbances. This indicates that when experimentally addressing different degrees of fragmentation, different temporal disturbance regimes, or the presence of bacterial dispersal networks, one should not draw conclusions on functional response by measuring a single characteristic like bacterial biomass alone. The same is true for observing the behavior of disturbed natural systems. Our study shows that it may be essential to analyze several ecosystem characteristics for understanding its overall stability under disturbances.

## Author Contributions

SK and TB developed the model. SK, TB, KF, MT, and FC designed the study. SK performed the simulations, analyzed the data, and wrote the manuscript. AW, TB, HH, MK, AM, LW, KF, MT, and FC provided consultation for the work and contributed significantly to the preparation of the manuscript. All authors approved its submission.

## Conflict of Interest Statement

The authors declare that the research was conducted in the absence of any commercial or financial relationships that could be construed as a potential conflict of interest. The reviewer SV and handling editor declare their shared affiliation.
