# Supplementary material for: Functional Resistance to Recurrent Spatially Heterogeneous Disturbances Is Facilitated by Increased Activity of Surviving Bacteria in a Virtual Ecosystem
Source: Front Microbiol. 2018 Apr 11;9:734. doi: 10.3389/fmicb.2018.00734 (PMC5904252; doi:10.3389/fmicb.2018.00734)
Supplement: Supplementary file 1 [file Data_Sheet_1.PDF]

Supplementary Material

Functional resistance to recurrent spatially heterogeneous disturbances is facilitated by increased activity of surviving bacteria in a virtual ecosystem

Sara König[1,2,4]*, Anja Worrich[2,3], Thomas Banitz[1], Hauke Harms[2,5], Matthias Kästner[3], Anja Miltner[3], Lukas Y Wick[2], Karin Frank[1,4,5], Martin Thullner[2], Florian Centler[2]

[1] UFZ - Helmholtz Centre for Environmental Research, Department of Ecological Modelling, Leipzig, Germany

[2] UFZ - Helmholtz Centre for Environmental Research, Department of Environmental Microbiology, Leipzig, Germany

[3] UFZ - Helmholtz Centre for Environmental Research, Department of Environmental Biotechnology, Leipzig, Germany

[4] University of Osnabrück, Institute for Environmental Systems Research, Osnabrück, Germany

[5] German Centre for Integrative Biodiversity Research (iDiv) Halle-Jena-Leipzig, Leipzig, Germany

*** Correspondence:**
Sara König
sara.koenig@ufz.de

1.1 Supplementary Figures

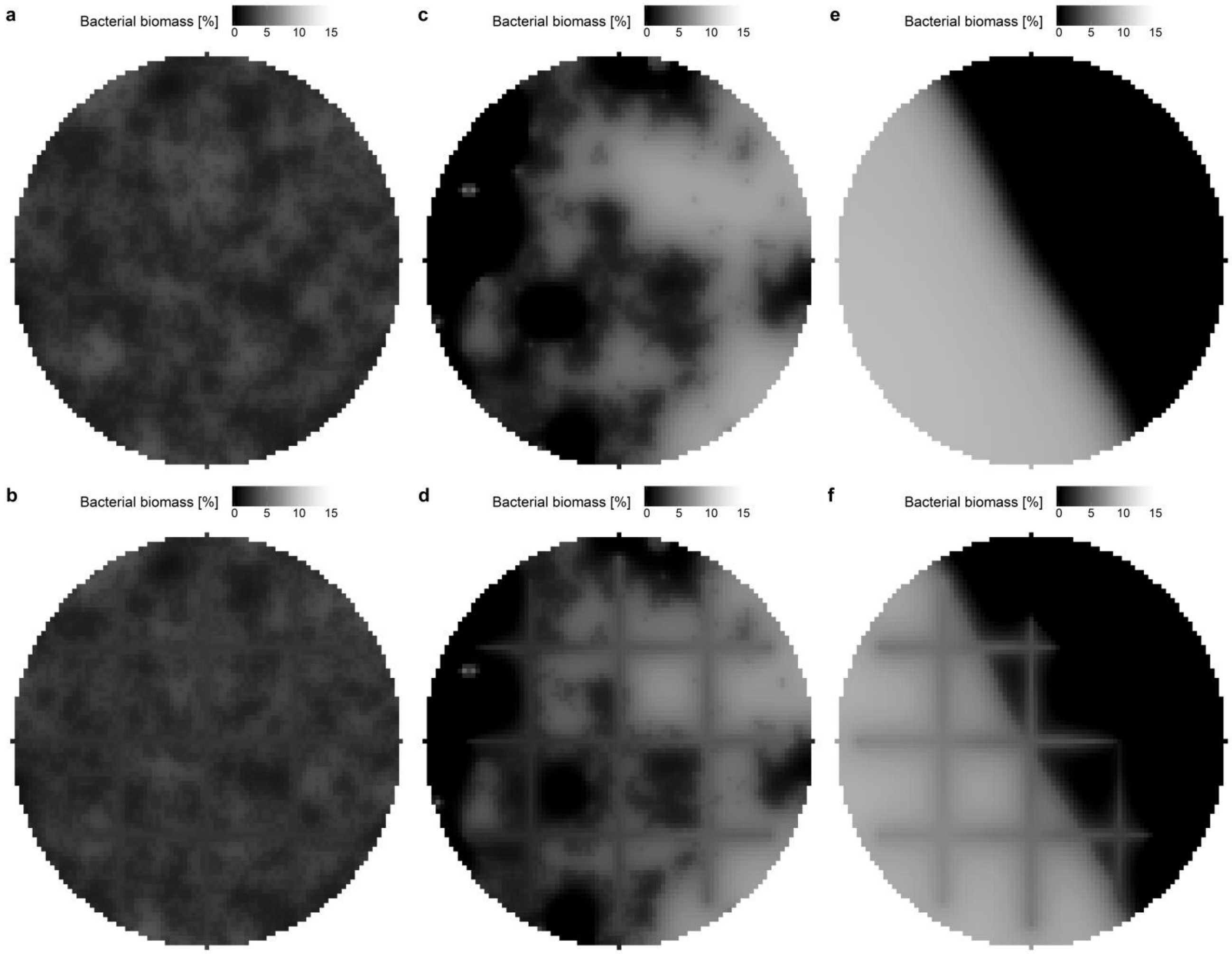

Supplementary Figure 1. Mean bacterial biomass in quasi-steady state without (a,c, e) and with dispersal networks (b, d, f) under recurrent disturbances with a disturbance return interval of 80 hours. Three different degrees of fragmentation of the disturbance pattern are shown: highly (a, b; H = -1), moderately (c, d; H = 0.5), and non-fragmented (e, f; H = 2).

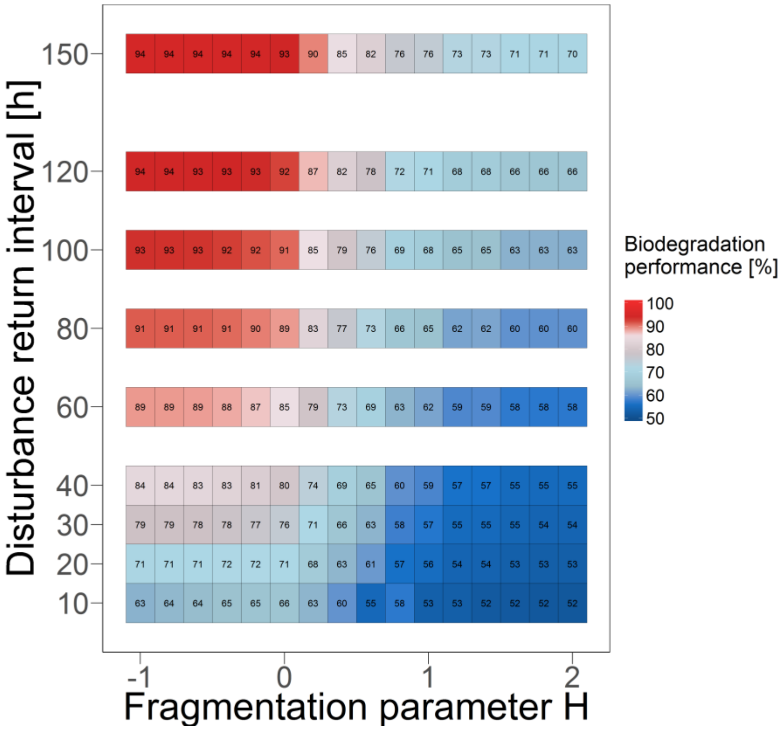

Supplementary Figure 2. Mean biodegradation performance in quasi-steady state as indicator for functional resistance for different disturbance return intervals and degrees of fragmentation. Boxes show mean values of 10 independent simulation runs with dispersal networks.

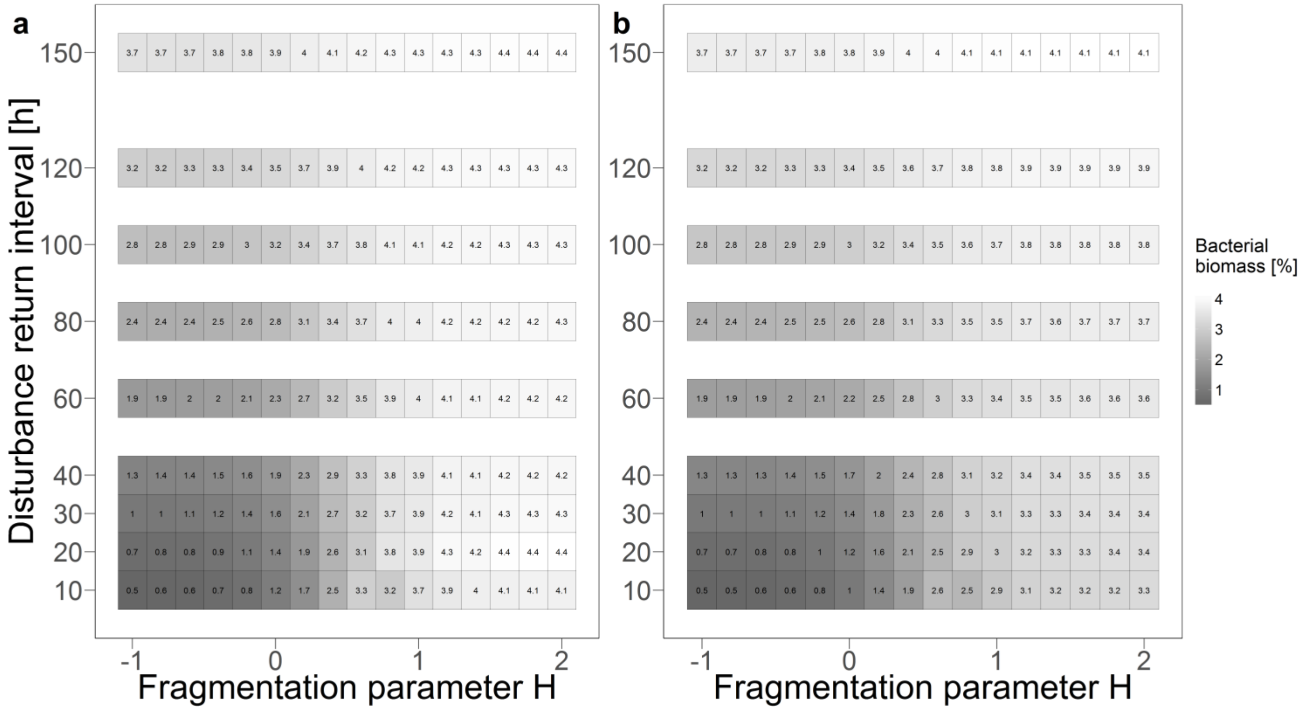

Supplementary Figure 3. Mean bacterial biomass in quasi-steady state for different disturbance return intervals and degrees of fragmentation. Boxes show mean values of 10 independent simulation runs without (a) and with (b) dispersal networks.

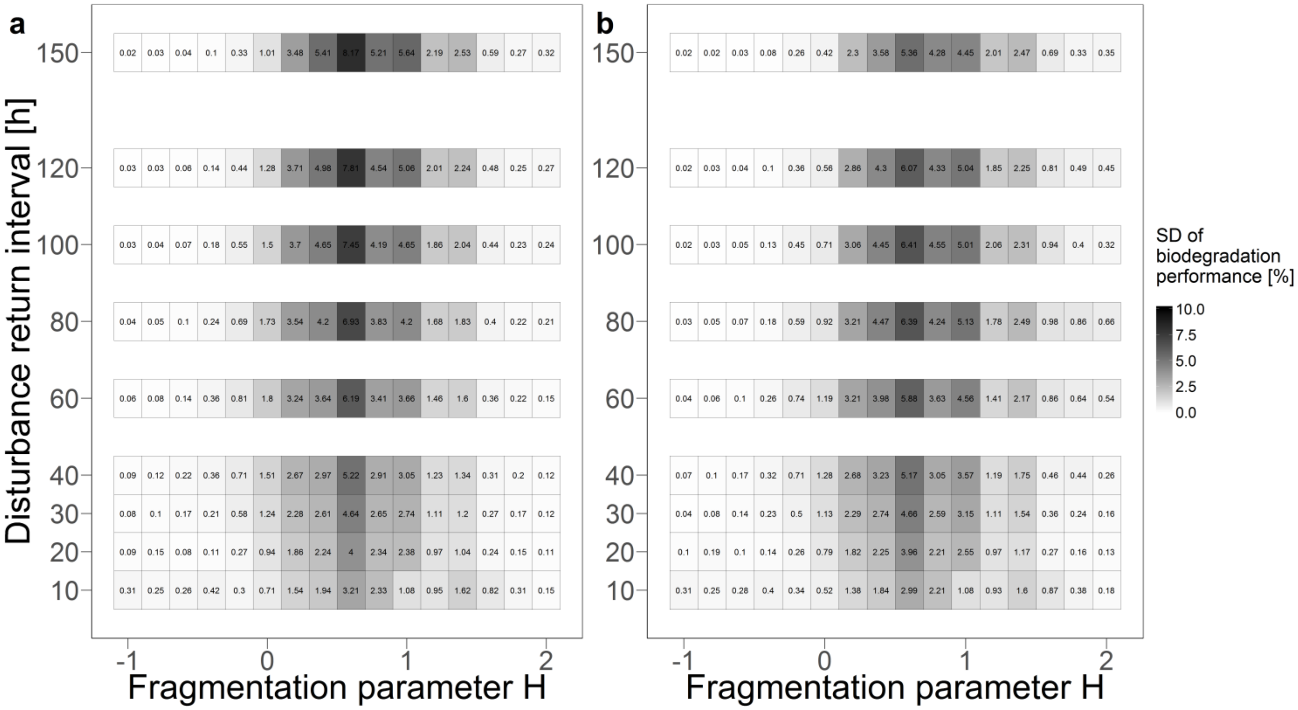

Supplementary Figure 4. Standard deviation of mean biodegradation performance in quasi-steady state for different disturbance return intervals and degrees of fragmentation. Boxes show SD values of 10 independent simulation runs without (a) and with (b) dispersal networks.

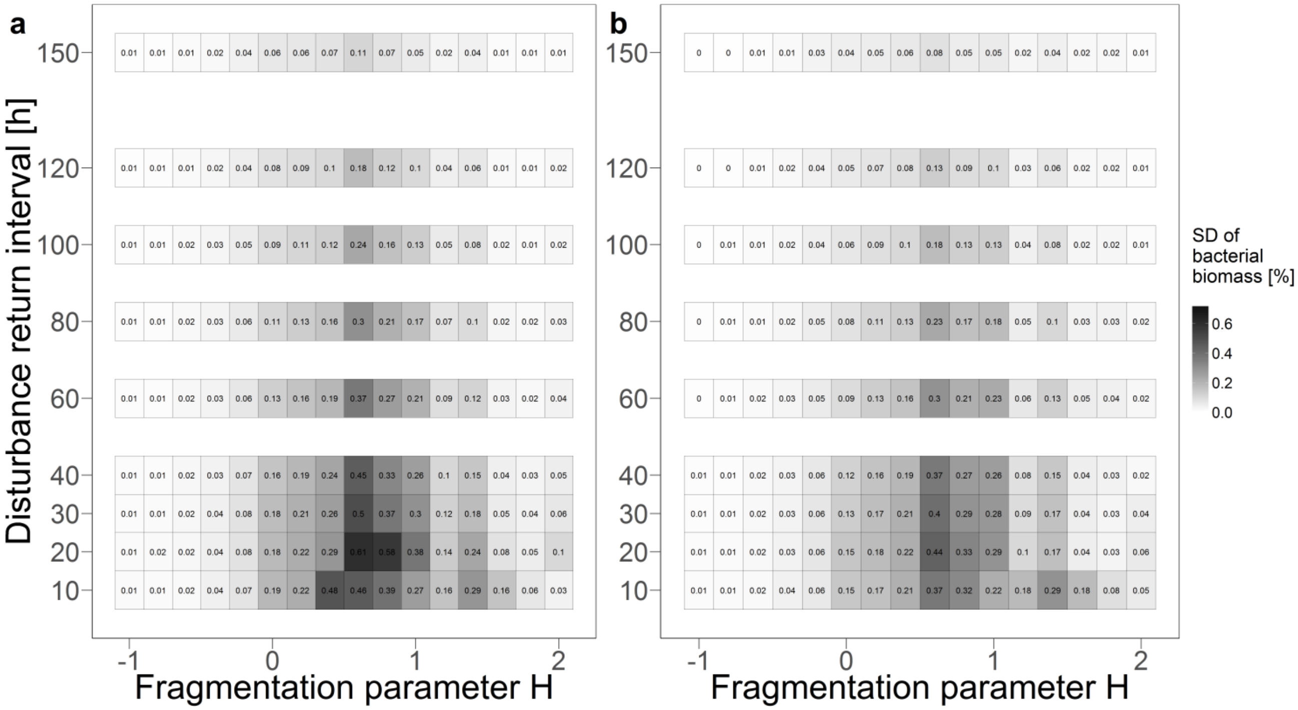

Supplementary Figure 5. Standard deviation of mean bacterial biomass in quasi-steady state for different disturbance return intervals and degrees of fragmentation. Boxes show SD values of 10 independent simulation runs without (a) and with (b) dispersal networks.

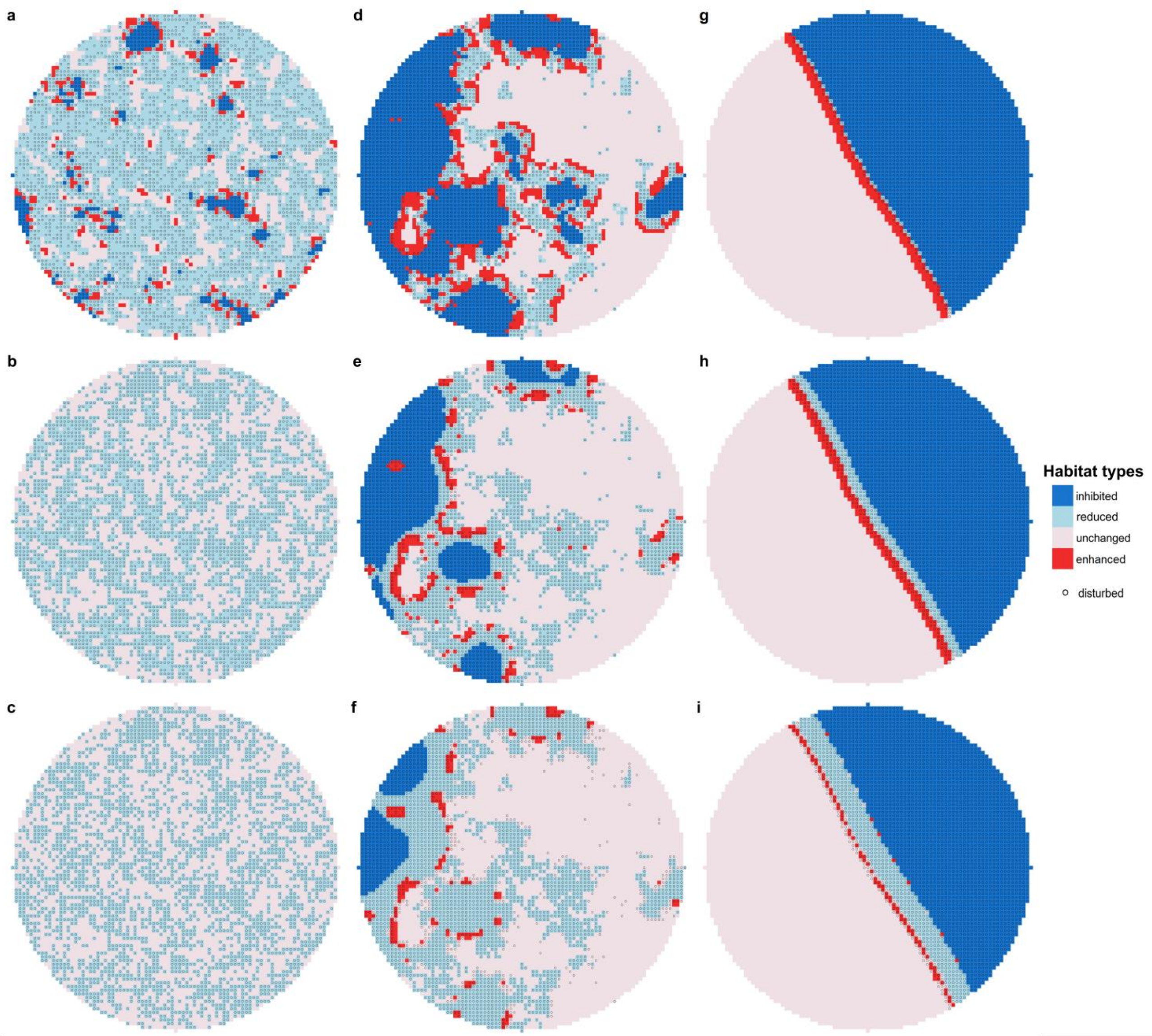

Supplementary Figure 6. Mean biodegradation performance in quasi-steady state without dispersal networks under recurrent disturbances with a disturbance return interval of 20 hours (a, d, g), 80 hours (b, e, h), and 150 hours (c, f, i). Three different degrees of fragmentation of the disturbance pattern are shown: highly (a, b, c; H = -1), moderately (d, e, f; H = 0.5), and non-fragmented (g, h, i; H = 2). Habitats within the disturbance area (50 % of total area) are marked with grey circles.

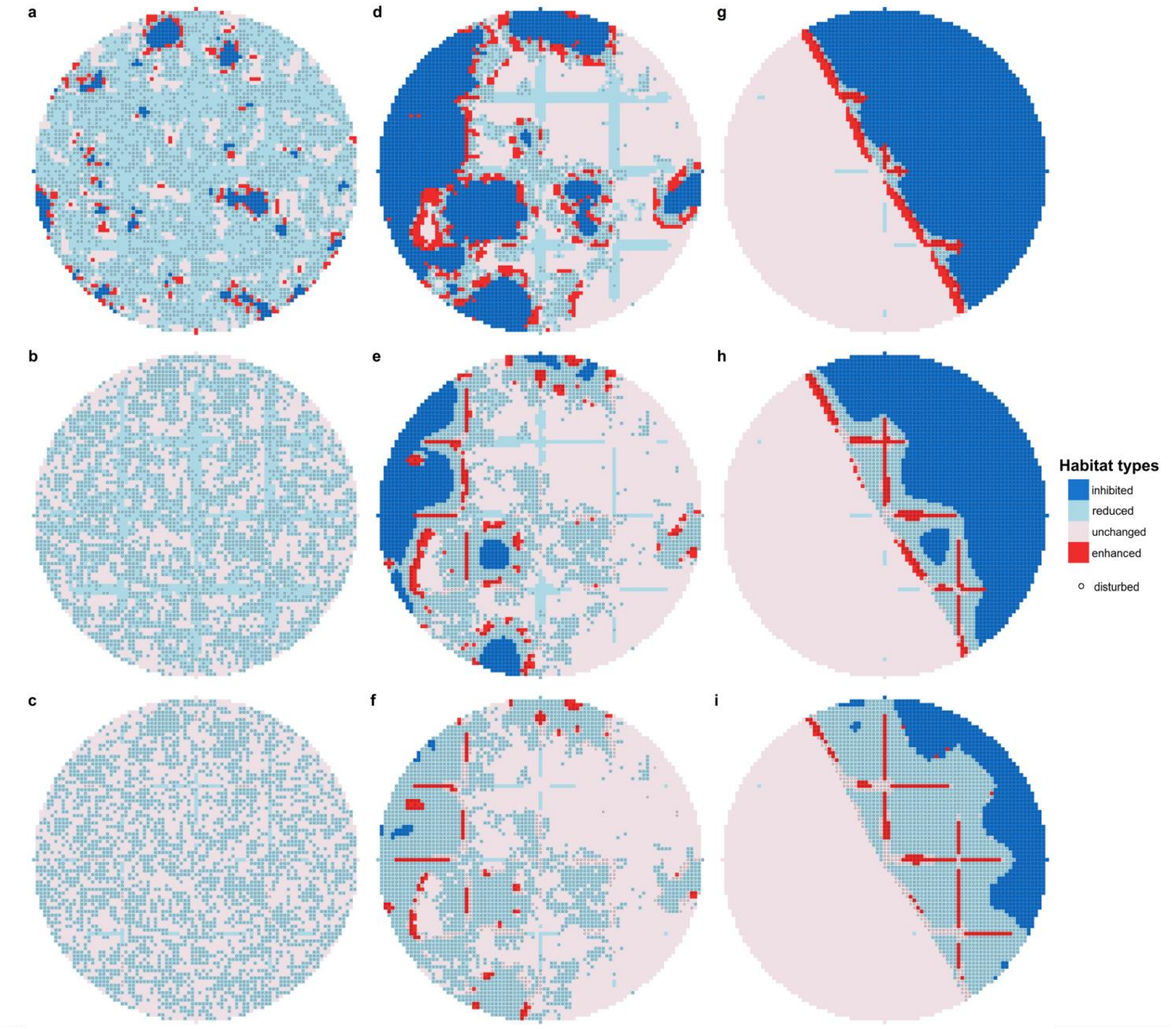

Supplementary Figure 7. Mean biodegradation performance in quasi-steady state with dispersal networks under recurrent disturbances with a disturbance return interval of 20 hours (a, d, g), 80 hours (b, e, h), and 150 hours (c, f, i). Three different degrees of fragmentation of the disturbance pattern are shown: highly (a, b, c; H = -1), moderately (d, e, f; H = 0.5), and non-fragmented (g, h, i; H = 2). Habitats within the disturbance area (50 % of total area) are marked with grey circles.

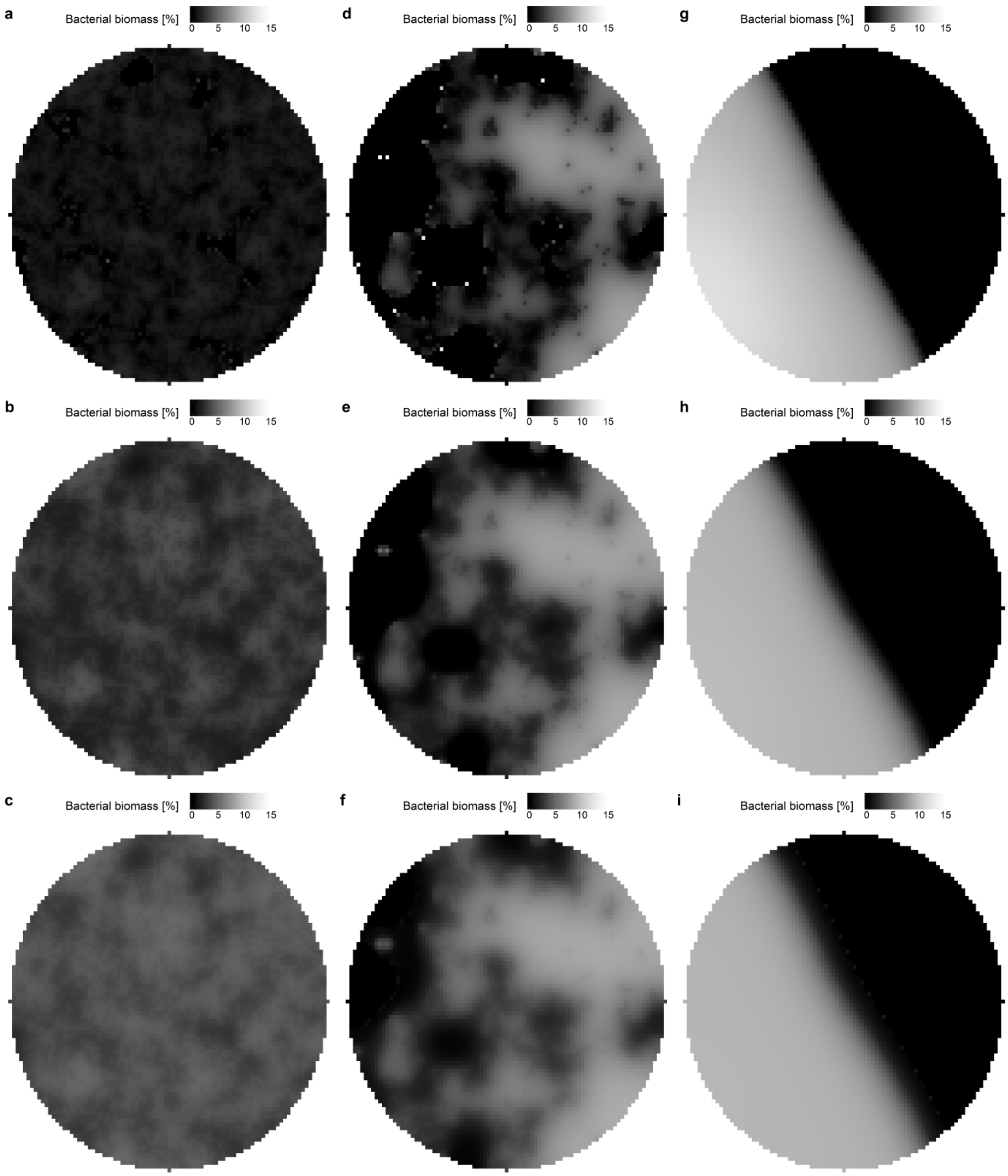

Supplementary Figure 8. Mean bacterial biomass in quasi-steady state without dispersal networks under recurrent disturbances with a disturbance return interval of 20 hours (a, d, g), 80 hours (b, e, h), and 150 hours (c, f, i). Three different degrees of fragmentation of the disturbance pattern are shown: highly (a, b, c; H = -1), moderately (d, e, f; H = 0.5), and non-fragmented (g, h, i; H = 2).

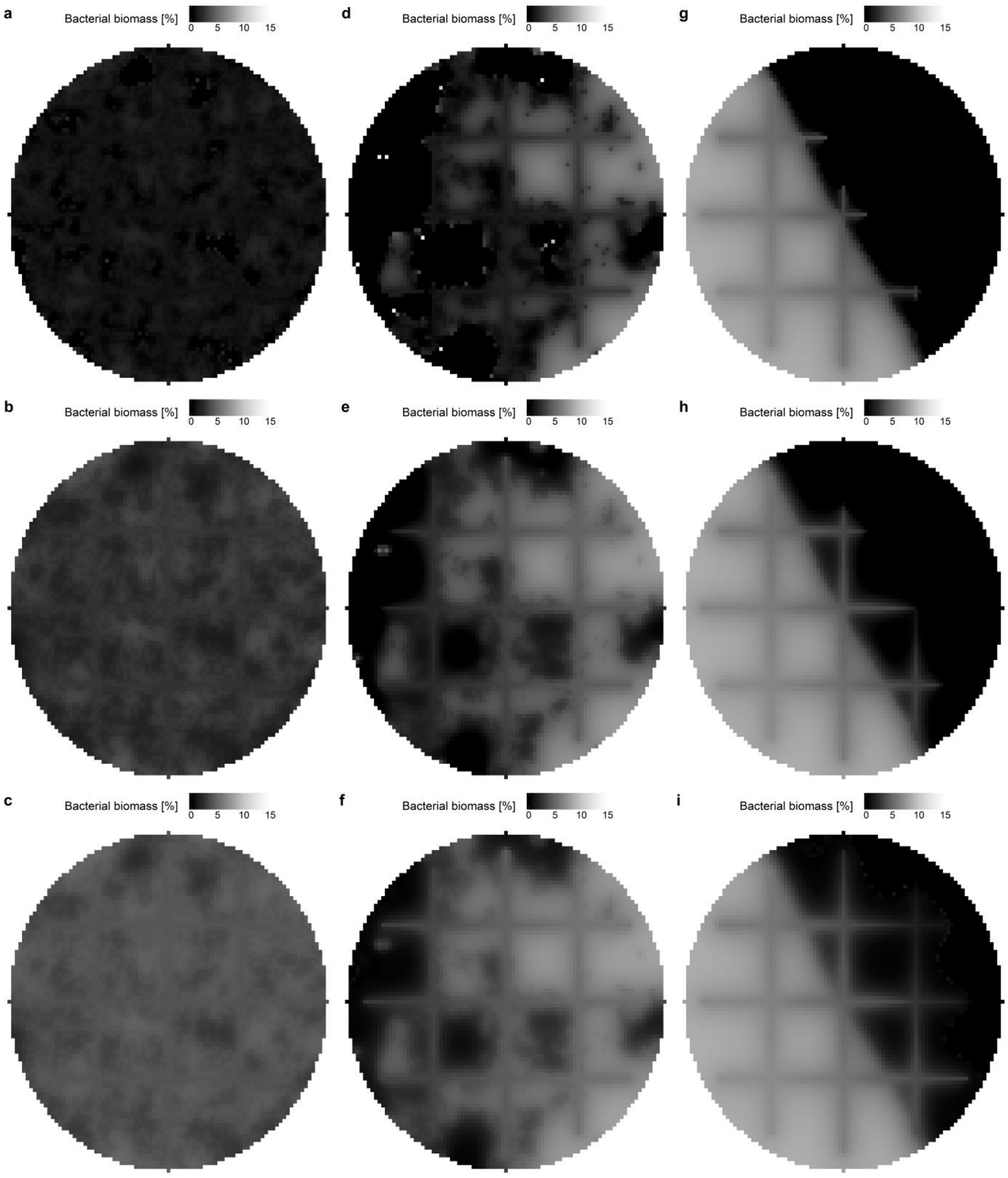

Supplementary Figure 9. Mean bacterial biomass in quasi-steady state with dispersal networks under recurrent disturbances with a disturbance return interval of 20 hours (a, d, g), 80 hours (b, e, h), and 150 hours (c, f, i). Three different degrees of fragmentation of the disturbance pattern are shown: highly (a, b, c; H = -1), moderately (d, e, f; H = 0.5), and non-fragmented (g, h, i; H = 2).

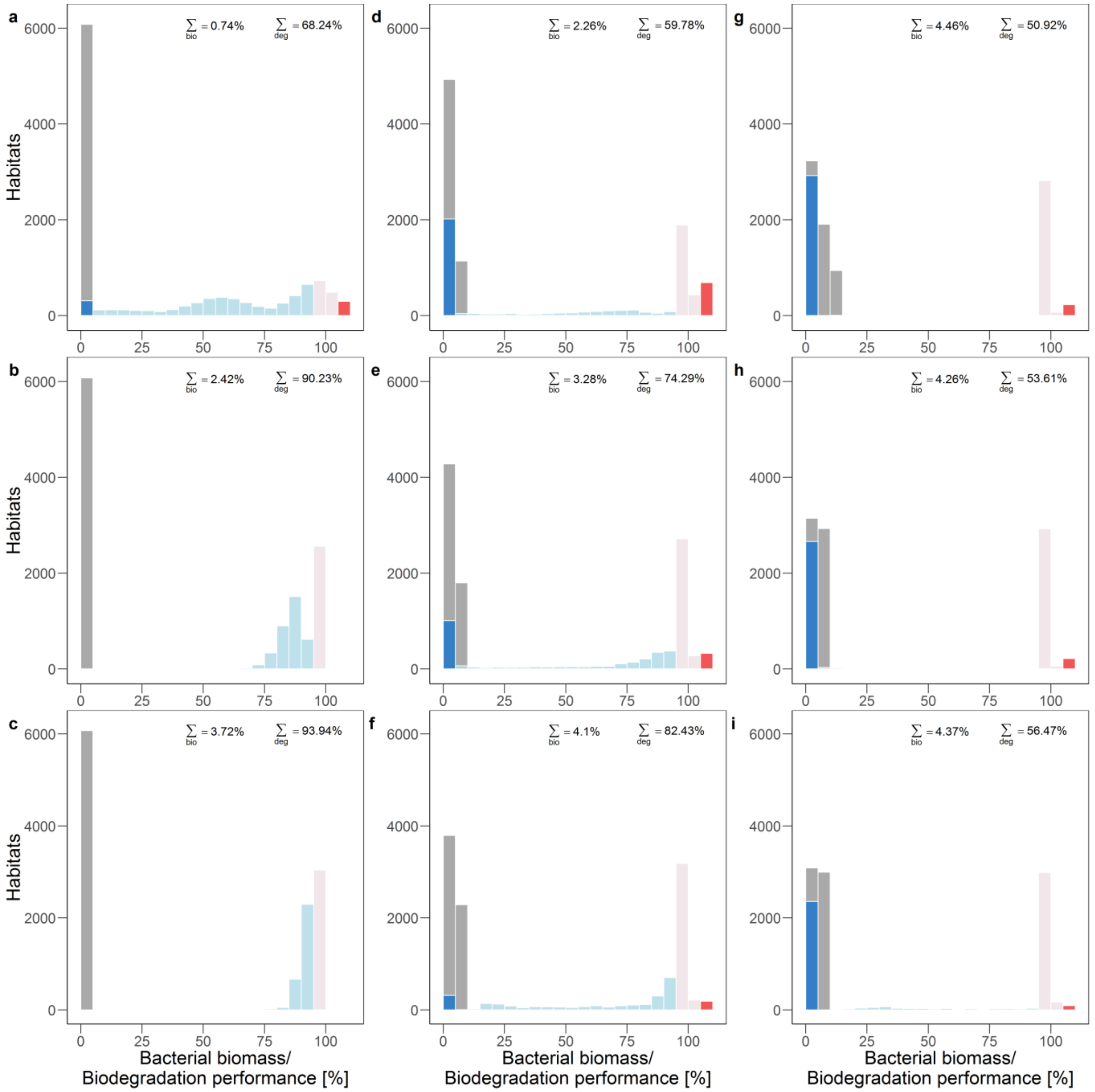

Supplementary Figure 10. Distribution of bacterial biomass (grey) and biodegradation performance (colors according to classification, cf Sec. 2.3) in quasi-steady state without dispersal networks under recurrent disturbances with a disturbance return interval of 20 hours (a, d, g), 80 hours (b, e, h), and 150 hours (c, f, i). Three different degrees of fragmentation of the disturbance pattern are shown: highly (a, b, c; H = -1), moderately (d, e, f; H = 0.5), and non-fragmented (g, h, i; H = 2).

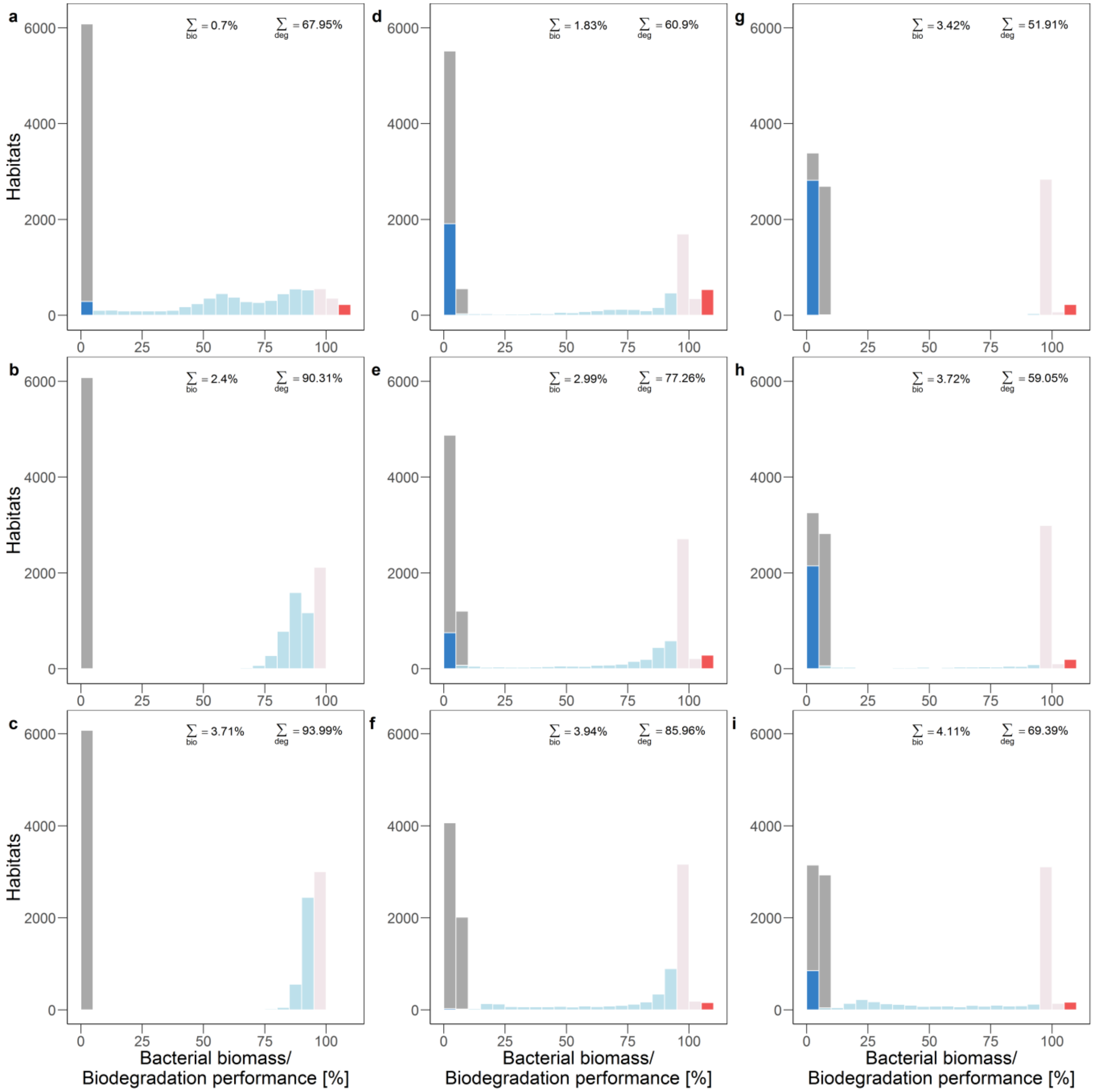

Supplementary Figure 11. Distribution of bacterial biomass (grey) and biodegradation performance (colors according to classification, cf Sec. 2.3) in quasi-steady state with dispersal networks under recurrent disturbances with a disturbance return interval of 20 hours (a, d, g), 80 hours (b, e, h), and 150 hours (c, f, i). Three different degrees of fragmentation of the disturbance pattern are shown: highly (a, b, c; H = -1), moderately (d, e, f; H = 0.5), and non-fragmented (g, h, i; H = 2).
